# Molecular and biochemical characterization of urease and survival of *Yersinia enterocolitica *biovar 1A in acidic pH *in vitro*

**DOI:** 10.1186/1471-2180-9-262

**Published:** 2009-12-17

**Authors:** Neeru Bhagat, Jugsharan S Virdi

**Affiliations:** 1 Microbial Pathogenicity Laboratory, Department of Microbiology, University of Delhi South Campus, Benito Juarez Road, New Delhi - 110 021, India

## Abstract

**Background:**

*Yersinia enterocolitica*, an important food- and water-borne enteric pathogen is represented by six biovars *viz*. 1A, 1B, 2, 3, 4 and 5. Despite the lack of recognized virulence determinants, some biovar 1A strains have been reported to produce disease symptoms resembling that produced by known pathogenic biovars (1B, 2-5). It is therefore imperative to identify determinants that might contribute to the pathogenicity of *Y. enterocolitica *biovar 1A strains. *Y. enterocolitica *invariably produces urease and the role of this enzyme in the virulence of biovar 1B and biovar 4 strains has been reported recently. The objective of this work was to study genetic organization of the urease (*ure*) gene complex of *Y. enterocolitica *biovar 1A, biochemical characterization of the urease, and the survival of these strains under acidic conditions *in vitro*.

**Results:**

The *ure *gene complex (*ureABCEFGD*) of *Y. enterocolitica *biovar 1A included three structural and four accessory genes, which were contiguous and was flanked by a urea transport (*yut*) gene on the 3' side. Differences were identified in *ure *gene complex of biovar 1A strain compared to biovar 1B and 4 strains. This included a smaller *ureB *gene and larger intergenic regions between the structural genes. The crude urease preparation exhibited optimal pH and temperature of 5.5 and 65°C respectively, and Michaelis-Menten kinetics with a K_m _of 1.7 ± 0.4 mM urea and V_max _of 7.29 ± 0.42 μmol of ammonia released/min/mg protein. The urease activity was dependent on growth temperature and growth phase of *Y. enterocolitica *biovar 1A, and the presence of nickel in the medium. The molecular mass of the enzyme was > 545 kDa and an isoelectric point of 5.2. The number of viable *Y. enterocolitica *biovar 1A decreased significantly when incubated at pH 2.5 for 2 h. However, no such decrease was observed at this pH in the presence of urea.

**Conclusions:**

The *ure *gene cluster of biovar 1A strains though similar to biovar 1B and 4 strains, exhibited important differences. The study also showed the ability of biovar 1A strains of *Y. enterocolitica *to survive at highly acidic pH *in vitro *in the presence of urea.

## Background

*Yersinia enterocolitica*, an important food- and water-borne human enteropathogen is known to cause a variety of gastrointestinal problems. Most commonly, it causes acute diarrhea, terminal ileitis and mesenteric lymphadenitis [1]. Long-term sequelae following infection include reactive arthritis and erythema nodosum [1]. Blood transfusion associated septicemia due to *Y. enterocolitica *has been reported to have high mortality [2]. Currently, *Y. enterocolitica *is represented by six biovars (1A, 1B, 2, 3, 4 and 5) and more than 30 distinct serovars. The virulence of known pathogenic biovars namely 1B and 2-5 is attributed to pYV (*p*lasmid for *Yersinia v*irulence) plasmid [3] and chromosomally borne virulence factors [4].

The biovar 1A strains however lack pYV plasmid and have generally been regarded as avirulent. But several clinical, epidemiological and experimental evidences indicate their potential pathogenicity [5]. Some biovar 1A strains have been reported to produce disease symptoms resembling that produced by pathogenic biovars [6,7]. These have been implicated in nosocomial [8] and food-borne [9] outbreaks and isolated from extra-intestinal sites [10]. The biovar 1A strains also invade epithelial cells [11,12], resist killing by macrophages [13] and carry virulence-associated genes such as *ystB *(enterotoxin), *inv *(invasin), *myfA *(fimbriae), *hreP *(subtilisin/kexin-like protease) and *tccC *(insecticidal-toxin like complex) [5,14]. In the past, enterotoxin has been thought to be the only major virulence factor produced by biovar 1A strains. Recently insecticidal-toxin complex [15] and flagella [16] have been identified as virulence factors of *Y. enterocolitica *biovar 1A strains. However the exact mechanisms underlying the pathogenesis by biovar 1A strains remains unclear and there is need to investigate the role of other putative virulence factors.

Urease (urea amidohydrolase; EC 3.5.1.5) has been implicated to play a role in the pathogenesis of many bacteria such as *Helicobacter pylori*, *Proteus mirabilis *and *Brucella abortus *[17-19]. *Y. enterocolitica *invariably produces urease which has been reported to enable biovar 1B and biovar 4 strains to survive in the acidic environment of the stomach [20,21]. However, the role of urease in the survival of biovar 1A strains has not been investigated. The objective of this study was to determine the genetic organization of urease (*ure*) gene cluster, factors affecting urease activity, and the survival of biovar 1A strain of *Y. enterocolitica *in acidic pH *in vitro*.

## Methods

### Bacterial strains and growth conditions

*Y. enterocolitica *biovar 1A (serovar O:6,30) isolated from the stools of a diarrheic patient and deposited with *Yersinia *National Reference Laboratory and WHO Collaborating Center, Pasteur Institute (Paris) under reference number IP27403 was used to characterize *ure *gene complex and the enzyme urease. The details of other *Y. enterocolitica *strains used in this study namely serovars, source of isolation, country of origin, reference laboratory accession numbers and clonal groups have been reported previously [22]. *Y. enterocolitica *8081 (bioserovar 1B/O:8) was obtained from M. Skurnik (Haartman Institute, Helsinki, Finland). *Y. enterocolitica *IP26329 (bioserovar 2/O:9), IP26249 (bioserovar 2/O:5,27), and IP134 (bioserovar 4/O:3) were obtained from E. Carniel (*Yersinia *National Reference Laboratory and WHO Collaborating Center, Pasteur Institute, France). All strains were grown overnight at 28°C in Luria broth (HiMedia, Mumbai, India).

### DNA extraction, primers and Polymerase Chain Reaction

Genomic DNA was isolated from overnight grown cultures using DNeasy tissue kit (Qiagen GmbH) as reported earlier [14].

Urease gene sequences of *Y. enterocolitica *biovar 1B and biovar 4 with GenBank accession numbers L24101[23] and Z18865[24] respectively were used to design primers U1 and U2 using PrimerSelect 5.03 software (DNASTAR Inc., Madison, USA) such that the structural genes (*ureA, ureB, ureC*) may be amplified as one amplicon. As these primers failed to consistently amplify the *ureABC *region of biovar 1A strains, primers for amplification of each of the structural genes separately were designed from the following sequences in the database (accession numbers are given in parentheses): *Y. enterocolitica *biovar 1B (L24101, AM286415), *Y. enterocolitica *biovar 4 (Z18865), *Y. aldovae *(AY363680), *Y. bercovieri *(AY363681), *Y. frederiksenii *(AY363682), *Y. intermedia *(AY363683), *Y. kristensenii *(AY363684), *Y. mollaretii *(AY363685), *Y. rohdei *(AY363686), *Y. pestis *(AE017042, AL590842, AE009952, AF095636) and *Y. pseudotuberculosis *(U40842, BX936398). These sequences were also used to design primers for *ure *accessory (*ureE*, *ureF*, *ureG*, *ureD*) and urea transport (*yut*) genes. The most conserved regions for each of the genes were identified using MegAlign (DNASTAR) or ClustalW version 1.83 (accessible at http://www.ebi.ac.uk/tools/clustalW).

**Figure 1:**
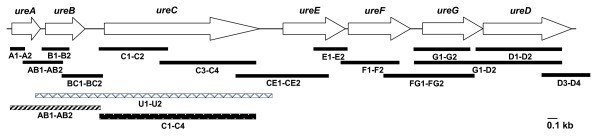
**Organization of *ure *gene cluster of *Y. enterocolitica *biovar 1A**. Primers used for amplification of structural and accessory genes, and the intergenic regions thereof are indicated.

Primer pairs - ureA1-ureA2 (for *ureA*), ureB1-ureB2 (for *ureB*), ureC1-ureC2 (for *ureC*), ureCE1-ureCE2 (for *ureC-ureE *and *ureE*), ureF1-ureF2 (for *ureF*), ureG1-ureG2 (for *ureG*), ureD1-ureD2 (for *ureD*) and yut1-yut2 (for *yut*) were designed from the conserved regions (Fig. 1) with PrimerSelect software. The sequences of the amplicons thus obtained (with strain IP27403) were used subsequently to design primers for the intergenic regions and a remaining part of the *ureC *gene. The intergenic regions between *ureA*-*ureB*, *ureB*-*ureC*, *ureE*-*ureF*, *ureF*-*ureG *and *ureD*-*yut *were amplified using primer pairs - ureAB1-ureAB2, ureBC1-ureBC2, ureE1-ureE2, ureFG1-ureFG2 and ureD3-ureD4 respectively and part of *ureC *gene by ureC3-ureC4. As *ureD *could not be amplified in biovar 1A strain with ureD1-ureD2, another primer pair ureG1-ureD2 was used for amplification of the *ureG*-*ureD *intergenic region and *ureD *gene. The primers were synthesized from Microsynth or Sigma Genosys. The details of the PCR primers and the target genes are given in Table 1.

**Table 1:** PCR amplification of urease structural (*ureA*, *ureB*, *ureC*) and the accessory (*ureE*, *ureF*, *ureG, ureD*) genes and the intergenic regions thereof, in *Y. enterocolitica *biovar 1A strain.

Primer	Sequence (5' - 3')	Target	Accession no.	Region amplified	Amplicon length (bp)	PCR conditions (°C, s)*		
						
						Den	Ann	Ext
U1 U2	G**C**AGCCGTTTGGTCAC**G**G CT**A**TGCCAC**G**CATCCCG**A**CC	*ureA-ureC*	DQ350880 AM286415 Z18865	275...2896 1075847...1078426 325...2907	**2622** 2580 2582	94, 60	62.0, 110	72, 110
								
ureA1 ureA2	GGAGGGCTTATGCAGCTCACCCCAAG TTGCCATCTCTGGCCCCTTCCA	*ureA*	DQ350880 AM286415 Z18865	1...161 1075573...1075733 51...211	**161** 161 161	94, 60	61.4, 60	72, 60
								
ureAB1 ureAB2	CAATGGAAGGGGCCAGAGATGG GTAAGCCGCAGCACGGTCAAACTC	*ureA-ureB*	DQ350880 AM286415 Z18865	137...579 1075709...1076210 187...688	**443** 502 502	94, 60	60.3, 60	72, 60
								
ureAB3 ureAB4	GCAGCTCACCCCAAGAGAAGTTGA AATTTGAGGCATCTGTCGCTCCTT	*ureA-ureB*	DQ350880 AM286415 Z18865	12...1015 1075584...1076608 62...1086	**1004** 1025 1025	95, 60	56.9, 110	72, 60
								
ureB1 ureB2	**A**TTGCAGAGGAT**T**AAAGCATGAGC AGCGGAACTTCGGTTTCATCACC	*ureB*	DQ350880 AM286415 Z18865	349...650 1075920...1076281 398...759	**302** 362 362	94, 60	60.0, 60	72, 60
								
ureBC1 ureBC2	TGCGGCTTACGGAAAAAGGCTGAATA GCCGAGAAATTTGAGGCATCTGTCG	*ureB*-*ureC*	DQ350880 AM286415 Z18865	570...1022 1076201...1076615 679...1093	**453** 415 415	94, 60	60.3, 60	72, 60
								
ureC1 ureC2	AAAGGAGCGACAGATGCCTCAAA GAAACCTGAATATCCATTTCATCCGCCAT	*ureC*	DQ350880 AM286415 Z18865	991...1749 1076584...1077342 1062...1823	**759** 759 762	94, 60	63.2, 60	72, 60
								
ureC3 ureC4	GGCTATAAAGTTCACGAAGACTG CAAAGAA**A**TAGCG**C**TGGTTCA	*ureC*	DQ350880 AM286415 Z18865	1661...2717 1077254...1078310 1735...2791	**1057** 1057 1057	94, 60	52.9, 60	72, 60
								
ureC1 ureC4	AAAGGAGCGACAGATGCCTCAAA CAAAGAAATAGCGCTGGTTCA	*ureC*	DQ350880 AM286415 Z18865	991...2717 1076584...1078310 1062...2791	**1727** 1727 1730	94, 60	50.0, 60	72, 120
								
ureCE1 ureCE2	GCGCTGGATGACGGTGTGAAAGAG ATGTAAGCCGGAGCCATGAGGTTC	*ureC*-*ureE*, *ureE*	DQ350880 AM286415	2504...3552 1078097...1079082	**1019** 986	94, 60	61.0, 60	72, 60
								
ureE1 ureE2	ACCATGATGGATTCCGTGATGAGA GTGAAGGCCCCGACCGGCAGTACG	*ureE-ureF*	DQ350880 AM286415	3364...3734 1078894...1079270	**371** 377	95, 60	58.7, 60	72, 60
								
ureF1 ureF2	TGAATGCATCAGATCTGATTCGTA ACATCCACAATAGGGACATAAGA	*ureF*	DQ350880 AM286415	3668...4304 1079204...1079840	**637** 637	95, 60	50.0, 60	72, 60
								
ureFG1 ureFG2	CAATATGGCGTGGCGATGACAAT CCACCGGGCCACCAATACCAA	*ureF-ureG*	DQ350880 AM286415	4132...4535 1079668...1080070	**403** 401	95, 60	55.7, 60	72, 60
								
ureG1 ureG2	GAATAGCCATTCAACCGATAAAC CGCATAATCATATCCACCAAC	*ureG*	DQ350880 AM286415	4474...5091 1080009...1080626	**618** 618	95, 60	51.3, 60	72, 60
								
ureG1 ureD2	GAATAGCCATTCAACCGATAAAC TTCCGGC**A**ATGTCACACC**G**A**G**AAT	*ureG-ureD, ureD*	DQ350880 AM286415	4474...6099 1080009...1081634	**1626** 1626	95, 60	50.4, 60	72, 120
								
ureD1 ureD2	AGCCAGAATATCGTGGAAACTCCT TTCCGGC**A**ATGTCACACC**G**A**G**AAT	*ureD*	DQ350880 AM286415	5146...6099 1080681...1081634	**954** 954	95, 60	50.0, 60	72, 60
								
ureD3 ureD4	TTGTTAACCCCCAAAGAGCATCAT CTGCCGGATTCCCTTCGCCATAG	*ureD*-*yut*	DQ350880 AM286415	5884...6416 1081419...1081950	**533** 532	95, 60	58.0, 60	72, 60
								
Yut1 Yut2	CGCGGCTGTGCTCAAGTC GTGCTGGCATCACATCTTTATTAGG	*yut*	AM286415	1081851...1082745	895	95, 60	50.0, 60	72, 60

The primer details and the PCR conditions used are given.

DQ350880:*Y. enterocolitica *IP27403 (bioserovar 1A/O:6,30); AM286415: *Y. enterocolitica *8081 (bioserovar 1B/O:8); Z18865: *Y. enterocolitica *6471/76 (bioserovar 4/O:3)

Nucleotides sequences in bold are different in biovar 1A strain (DQ350880)

*PCRs were performed with initial denaturation step of 94°C for 10 min, 30 cycles each of denaturation (Den), annealing (Ann) and extension (Ext) as indicated and a final extension of 10 min at 72°C

PCRs for *ure *structural and accessory genes, intergenic regions and the *yut *gene were performed using a thermal cycler (MyCycler, Bio-Rad). The 25 μl PCR reaction mixture contained 100 ng of genomic DNA, 2.5 μl of 10 × *Taq *buffer containing 1.5 mM MgCl_2_, 2.5 μl of 2 mM dNTP, 25 pmol of each primer, and 2 U of *Taq *DNA polymerase (New England BioLabs). The details of the conditions used for amplification are given in Table 1. After amplification, 10 μl of the PCR product was resolved in 2% agarose gel in 1 × Tris-acetate-ethylenediaminetetraacetic acid (TAE) buffer (40 mM Tris-HCl, 20 mM acetic acid, 1 mM EDTA, pH 8.0) at 70 V for 2 h. The gels were stained with ethidium bromide (0.5 μg/ml) and photographed under UV-transillumination in a gel documentation system (Bio-Rad, CA). The 1 kb and 100 bp DNA ladders (New England BioLabs) served as molecular size markers.

### Sequencing of PCR amplicons, ORF analysis and phylogenetic relationships

The PCR amplicons obtained above using the genomic DNA of *Y. enterocolitica *biovar 1A (strain IP27403) were extracted, purified using QIAquick Gel extraction kit (Qiagen) and sequenced directly in one or both directions (Microsynth, Balgach, Switzerland or LabIndia, Gurgaon, India). The sequences were analyzed, edited and compiled using Editseq and MegAlign of DNASTAR. Homology searches for nucleotide and deduced amino acid sequences were carried out by BLASTN and BLASTP respectively. The multiple nucleotide and protein sequence alignments were performed by MegAlign or ClustalW. The percent identity and similarity were calculated using MatGAT 2.02 [25]. The theoretical molecular weight and isoelectric point (pI) of urease structural and accessory proteins were determined by EditSeq (DNASTAR).

The open reading frames (ORFs) in the compiled *ure *gene cluster were identified using GeneMark [26], GeneMark.hmm [27], FGENESB [28] and the NCBI ORF finder [29] programs. All ORFs were checked further for homology to known protein sequences using BLASTX.

The relationship of urease structural and accessory protein sequences of biovar 1A strain of *Y. enterocolitica *to sequences available in GenBank were determined by constructing phylogenetic trees with the program MEGA 4.0 using the neighbor-joining algorithm. Bootstrap value for each node of the tree was calculated over 1,000 replicate trees.

### PCR-Restriction fragment length polymorphism (PCR-RFLP) of urease genes

Primer pairs ureAB3-ureAB4 and ureC1-ureC4 were designed to amplify the 1,004 bp and 1,727 bp of *ureAB *and *ureC *genes respectively (Fig. 1). The biovar 1A strains were chosen such that each belonged to a different serovar, country, source of isolation, REP/ERIC-type [22] and VNTR01-type [30]. The PCR amplicon of *ureAB *was digested with *Hae*III and *Sau*96I while that of *ureC *was digested with *Rsa*I and *Sau*96I. The choice of the restriction enzymes was based on *in silico *restriction of the expected amplicons such that DNA fragments were amenable to separation by gel electrophoresis. Restriction enzymes were from New England BioLabs (*Rsa*I and *Hae*III) or Bangalore Genei (*Sau*96I). Ten microlitre of amplified DNA was digested with 2.5 U (*Hae*III and *Sau*96I) or 5 U (*Rsa*I) of restriction enzyme using appropriate buffer recommended by the manufacturer, in a total volume of 25 μl at 37°C overnight. The digested products were separated by electrophoresis in 2.5% agarose gel at 50 V for 5 h in TAE buffer. 100 bp ladder (New England BioLabs) was used as the molecular size standard. The gel was stained with ethidium bromide and examined under UV transillumination.

### Growth and preparation of cell free extract

*Y. enterocolitica *strain IP27403 was grown overnight at 28°C in 20 ml LB medium with shaking at 200 rpm. Cells were collected by centrifugation (9,000 × *g*, 10 min, 4°C), washed twice, and resuspended to 1.5 × 10^8 ^CFU/ml equivalent to 0.5 McFarland standard (A_600 _= 0.1). These were diluted to 1.0 × 10^6 ^CFU/ml and 50 μl of this suspension was inoculated into 50 ml of fresh LB medium, and incubated further (28°C, shaking at 200 rpm). Samples were withdrawn at different time intervals up to 78 h and diluted in 20 mM sodium phosphate buffer (pH 7.0). 0.1 ml of the appropriate dilution was plated, in triplicate, on Luria agar and incubated overnight at 28°C. The number of viable bacteria was recorded at different intervals and CFU/ml was calculated. The log_10_CFU/ml was plotted against incubation time (in h).

For preparing lysate, cells grown in 50 ml LB medium were harvested by centrifugation, washed twice and resuspended in 2.5 ml of 20 mM sodium phosphate buffer (pH 7.0). Cells were disrupted by sonication with three cycles (2 s "pulse on" and 2 s "pulse off" for 2 min) at 25% intensity with Vibra-Cell (Sonics). The cell lysate was centrifuged at 18,000 × *g *for 30 min at 4°C to obtain cell-free extract. The supernatant was transferred to pre-chilled microcentrifuge tubes and used immediately for determination of urease activity. Protein concentration was estimated by Bradford [31] method using bovine serum albumin (Sigma) as standard.

### Urease assay

Urease activity in the cell extract was assayed by measuring release of ammonia from urea in the phenol-hypochlorite assay [32]. Briefly, extract containing 2 μg of protein was added to 100 mM citrate buffer (pH 5.5) containing 50 mM urea in 200 μl of final volume. The mixture was incubated at 37°C for 15 min. A similar volume of the extract boiled for 10 min served as negative control. The reaction was terminated by the addition of 1.5 ml of solution containing 1% phenol and 0.005% sodium nitroprusside; this was followed by the addition of 1.5 ml solution containing 0.5% (w/v) NaOH and 0.044% (v/v) NaClO, and the contents were mixed well. Following incubation at 37°C for 30 min, the absorbance was measured at 625 nm using a spectrophotometer (UV-1700 Pharmaspec; Shimadzu Scientific Instruments Inc., Columbia, Md.). Assays were carried out in triplicate and the amount of the ammonia released per minute was determined. The quantity of ammonia (in nmol) released was calculated from the calibration curve obtained from appropriate dilutions of freshly prepared NH_4_Cl solution, which was determined to be linear between 20-500 nmol. Data are presented as specific activity of urease, defined as μmol of NH_3_/min/mg of protein. Stated values are the mean ± standard deviation of triplicate determinations.

### Biochemical characterization

The optimum pH for urease was determined by measuring activity at pH 1.5 to 7.5. The assays were carried out in 20 mM sodium phosphate (for pH 1.5, 2.5, 5.5, 6.0, 6.5, 7.0 and 7.5) and 100 mM citrate (for pH 3.0, 3.5, 4.0 and 5.5) buffers. The optimum temperature for urease was determined by incubating the extract containing enzyme with substrate at different temperatures (18-75°C) in the phenol-hypochlorite assay described above. The kinetic data (K_m _and V_max_) of urease were calculated from Lineweaver-Burk plot of the initial rate of hydrolysis of urea in citrate buffer (100 mM, pH 5.5). To determine the effect of growth temperature and growth phase on urease activity, extracts were prepared from cells grown in shaking at 28°C and 37°C in LB medium for different time intervals. To determine the effect of urea and nickel on production of urease, medium was supplemented with urea (16.7 mM) or NiCl_2 _(up to 200 μM).

### Native and SDS PAGE

Cell-free extracts from different biovars of *Y. enterocolitica *were electrophoresed on non-denaturing polyacrylamide gel [33]. Briefly, extract containing *ca*. 100 μg of protein was mixed with 1× tracking dye and loaded on 5% resolving gel in 380 mM Tris-HCl (pH 8.8) with 4% stacking gel in 63 mM Tris-HCl (pH 6.8) in a mini-Protein III apparatus (Bio-Rad). Samples were electrophoresed with Tris-Glycine (pH 8.4) as the running buffer at 70 V for 2 h at 4°C. The gel was removed and equilibrated with 5-10 changes of solution containing 0.02% cresol red and 0.1% EDTA until the entire gel turned yellow. After draining the solution, gel was flooded with 1.5% (w/v) solution of urea. The pink bands of urease were recorded by scanning (UMAX Astra 3600). Urease from jack bean (Sigma) was used as the marker.

SDS-PAGE was performed as per standard protocol [34]. Briefly, extract containing 25 μg of protein was boiled in reducing Laemmli sample buffer and separated on 12% polyacrylamide gel.

### Isoelectric focusing (IEF)

IEF of the cell extract was carried out in 6% polyacrylamide gel containing 2% ampholyte of pH 3-10 (Biolyte Ampholyte, Bio-Rad). 3-5 μl of extract containing *ca*. 20-25 μg of protein was loaded on the gel and focused at 4°C using a Mini IEF cell (Bio-Rad) according to the manufacturer's instructions. After focusing, the gel was equilibrated with a solution containing 0.02% cresol red and 0.1% EDTA. Urease bands were visualized by superimposing the gel with Whatman No. 1 filter paper presaturated with cresol red-EDTA solution containing 1.5% urea. Urease appeared as pink band against a yellow background. Broad range IEF standard with pI 4.45-9.6 (Bio-Rad) was used as the pI marker to determine the isoelectric point of the urease.

### Survival of *Y. enterocolitica *in acidic pH *in vitro*

The *in vitro *survival of *Y. enterocolitica *was performed by slight modification of the method reported earlier [35]. Briefly, ten microlitre of the bacterial suspension was added to 1 ml of 20 mM sodium phosphate (for pH 2.5 and 7.0) or 100 mM citrate (for pH 4.0) buffer with or without 3.4 mM urea in 0.6% NaCl, and prewarmed to 37°C to give an initial count of *ca. *7.0 log_10_CFU/ml. The contents were mixed and incubated with shaking at 37°C for 2 h. At the end of the incubation, samples were removed and diluted serially in 20 mM sodium phosphate buffer (pH 7.0). 0.1 ml of an appropriate dilution was plated on LB agar to determine CFU/ml. At conclusion of each assay, the pH of the buffer was recorded. All assays were repeated at least thrice on separate occasions.

### Statistical analysis

The mean and the standard deviation for each data set were calculated using Microsoft Excel 2003 software (Microsoft Corporation, Redmond, Wash.). Statistical significance was calculated using unpaired *t *test (Sigma Stat version 3.5). *p *value < 0.05 was considered significant.

### Nucleotide sequence accession number

The nucleotide sequence data of *ure *gene complex and the *yut *gene reported in this paper have been deposited in GenBank database under accession numbers DQ350880 and EU527335 respectively.

## Results

### Characterization of urease genes

Primers U1 and U2 were designed to amplify the *ure *structural (*ureA*, *ureB*, *ureC*) genes of *Y. enterocolitica*. Although amplification was obtained with biovar 1B, 2 and 4 strains, these primers did not consistently amplify the *ure *structural genes of biovar 1A strains. Thus, new primers were designed to amplify each of the *ure *structural and accessory (*ureE*, *ureF*, *ureG*, *ureD*) genes separately, and the intergenic regions so as to encompass the entire urease gene cluster of biovar 1A strain. Amplicons of expected sizes were obtained for all genes except *ureB *and the intergenic regions namely *ureA*-*ureB*, *ureB*-*ureC *and *ureC*-*ureE *(Table 1). The sequences thus obtained were analyzed for homology with sequences available in databases, edited and combined to obtain 7,180 bp sequence of *ure *gene cluster of biovar 1A strain (See Additional file 1 for *ure *gene cluster sequence).

Seven ORFs were identified in the *ure *gene cluster of *Y. enterocolitica *biovar 1A strain and designated as *ureA*, *ureB*, *ureC*, *ureE*, *ureF*, *ureG *and *ureD *(Fig. 1) as in the *ure *gene complex of *Y. enterocolitica *8081 (biovar 1B, accession number AM286415). As with *Y. enterocolitica *8081, *yut *gene which encodes a urea transport protein was present downstream of the *ure *gene cluster. All ORFs had ATG as the start codon except *ureG *where the start codon was GTG. These ORFs were preceded by ribosome-binding consensus sequence. Although *ure *gene cluster of biovar 1A strain was broadly similar to that of biovar 1B and biovar 4 strains, differences were identified. These were - smaller *ureB *gene and *ureA*-*ureB *intergenic region and larger *ureB*-*ureC *and *ureC*-*ureE *intergenic regions in biovar 1A strain (Table 2). The size of *ureB *gene of *Y. enterocolitica *biovar 1A was identical to *ureB *of *Y. aldovae*, *Y. bercovieri*, *Y. intermedia*, *Y. mollaretii *and exhibited higher nucleotide sequence identity to these species than to *Y. enterocolitica *biovar 1B or 4. The stop codon of *ureG *overlapped with the start codon of *ureD *gene. The G + C content of the urease gene cluster was 49.76% which was typical of *Y. enterocolitica *with G + C content of 47.27%.

**Table 2:** Urease structural and accessory genes and the intergenic regions thereof, in *Y. enterocolitica *biovar 1A.

Gene/Intergene	Ye1A	Size (in bp) and % identity							
		
		YeO8	YeO3	Yers	Yps	Ype	Pl	Ei	Ka
**Structural**									
*ureA*	303	303 **98**	303 **98**	303 **89-96**	303 **91**	303 **91**	303 **77**	303 **76**	303 **63**
*ureB*	435	495 **83**	495 **83**	435-495* 78-89	435/477 **74-82**	435/477/528 **67-81**	390 **63**	423 **62**	321 **48**
*ureC*	1719	1719 **96**	1722 **94**	1719 **87-91**	1719 **86**	1719 **82-86**	1716 **75**	1719 **76**	1704 **61**
**Accessory**									
*ureE*	687	792/693 **85/97**	NA	681-720 **83-91**	696 **82**	696/705 **80-82**	597 **59**	678 **58**	477 **44**
*ureF*	687	687 **98**	NA	687 **85-90**	687 **85-86**	687 **86**	687 **66**	705 **65**	675 **48**
*ureG*	666	666/606 **99/90**	NA	666 **88-92**	663 **84**	663 **85**	636 **78**	630 **73**	618 **59**
*ureD*	984	978/984 **96/97**	NA	966-984 **84-90**	966 **81**	834/964/967 **71-82**	966 **64**	963 **62**	-
**Intergenic region**									
*ureAB*	54	53	53	53-65	10/52	0/10/52	91	46	9
*ureBC*	202	164	164	87-97/201-202*	89	89	67	42	0
*ureCE*	236	74/173	NA	133-204	294/295	286/295	74	57	9
*ureEF*	21	21	NA	20-24	21	21	0	0	1
*ureFG*	117	117/177	NA	58-102	125/126	126	52	14	8
*ureGD*	0	1/0	NA	0	0	0	0	6	-

Comparison with different *Yersinia *spp. and other bacteria.

The abbreviations correspond to following species with accession number(s) in parentheses. Ye1A: *Y. enterocolitica *bioserovar 1A/O:6,30 (DQ350880); YeO8: *Y. enterocolitica *bioserovar 1B/O:8 (L24101, AM286415); YeO3: *Y. enterocolitica *bioserovar 4/O:3 (Z18865); Yers included *Y. aldovae *(AY363680), *Y. bercovieri *(AY363681), *Y. frederiksenii *(AY363682), *Y. intermedia *(AY363683), *Y. kristensenii *(AY363684), *Y. mollaretii *(AY363685), *Y. rohdei *(AY363686); Yps: *Y. pseudotuberculosis *(U40842; CP000720; CP000950; BX936398); Ype: *Y. pestis *(CP000901, CP000308, AL590842, AE017042, CP000305, CP000668, AF095636); Pl: *Photorhabdus luminescens *(BX571866); Ei: *Edwardsiella ictaluri *(AY607844); Ka: *Klebsiella aerogenes *(M36068)

% identity is indicated in bold

0 indicates that the intergenic region had overlapping stop and start codons

*ureB gene size was 435 bp (*Y. aldovae*, *Y. bercovieri*, *Y. intermedia*, and *Y. mollaretii*), 441 bp (*Y. rohdei*), 468 bp (*Y. frederiksenii*) and 495 bp (*Y. kristensenii*); *ureBC *intergenic region of 201-202 bp was present in *Y. aldovae *and *Y. intermedia*

The comparison of *Y. enterocolitica *biovar 1A *ure *genes and the deduced amino acid sequences with that of *Yersinia *spp. and other bacteria are given in Tables 2 and 3 respectively. Besides *Yersinia *species, the homologies of *ure *genes (upto 76% identity) and their deduced amino acid sequences (upto 86% identity and 95% similarity) were significant with ureases from *Photorhabdus luminescens *and *Edwardsiella ictaluri*. The UreA, UreC and UreG proteins were most conserved among *Yersinia *spp. The estimated molecular weights, in Da, of the protein subunits were 11,048 (UreA), 15,854 (UreB), 61,026 (UreC), 25,507 (UreE), 25,040 (UreF), 24,181 (UreG) and 36,592 (UreD) (Table 3).

**Table 3:** Urease structural and accessory proteins of *Y. enterocolitica *biovar 1A (Ye 1A).

	Gene	Gene product (aa)	Mol. mass (Da)*	pI*	% identity/% similarity							
					
					YeO8	YeO3	Yers	Yps	Ype	Pl	Ei	Ka
**Structural subunits**												
UreA	*ureA*	100	11,048	5.29	99-100	100	97-100/100	100	100	79/95	86/95	60/82
UreB	*ureB*	144	15,854	9.06	84-85/85-86	85/86	84-99/85-99	86-94/88-97	78-94/79-97	60/72	61/73	36/47
UreC	*ureC*	572	61,026	5.64	99/100	95/97	97-99/99-100	97/99	93-97/95-99	83/91	86/94	58/73
**Accessory proteins**												
UreE	*ureE*	228	25,507	6.35	86-99	NA	91-95/95-97	94/97	92-93/96-97	55/69	50/70	27/39
UreF	*ureF*	228	25,040	6.41	100	NA	96-99/97-100	98/99	97-98/99	67/76	65/83	22/43
UreG	*ureG*	221	24,181	4.94	91-100	NA	98-100/99-100	96/97	96/97	86/91	86/91	54/71
UreD	*ureD*	327	36,592	6.61	93-98/95-99	NA	91-98/95-99	93/96	FS	64/77	59/71	-

Comparison with different *Yersinia *spp. and other bacteria.

The abbreviations correspond to following species with protein accession numbers for UreA, UreB, UreC, UreE, UreF, UreG and UreD in parentheses: Ye1A: *Y. enterocolitica *biovar 1A (ABC74582-ABC74585; ACA51855-ACA51857); YeO8: *Y. enterocolitica *O8 biovar 1B (AAA50994-AAA51000, CAL11049-CAL11055); YeO3: *Y. enterocolitica *O3 biovar 4 (CAA79314-AA79320); Yers included *Y. aldovae *(AAR15084-AAR15090); *Y. bercovieri *(AAR15092-AAR15098); *Y. frederiksenii *(AAR15100-AAR15106); *Y. intermedia *(AAR15108-AAR15114); *Y. kristensenii *(AAR15117-AAR15123); *Y. mollaretii *(AAR15126-AAR15132); *Y. rohdei *(AAR15135-AAR15141); Yps: *Y. pseudotuberculosis *(CAH22182-CAH22176, AAA87852-AAA87858, ACA67429-ACA67435); Ype: *Y. pestis *(ABG14357-ABG14363; CAL21284-CAL21289; AAS62666-AAS62671; AAM84812-AAM84817; ABG17479-ABG17485; ABP39996-ABP39990; AAC78632-AAC78638); Pl: *Photorhabdus luminescens *(CAE14464-CAE14470); Ei: *Edwardsiella ictaluri *(ABD93708-ABD93706, AAT42448-AAT42445); Ka: *Klebsiella aerogenes *(AAA25149-AAA25154); NA: Not available; FS: frameshift mutation

* Theoretical molecular mass and pI were determined with DNASTAR

Phylogenetic analysis of urease structural and accessory proteins of *Y. enterocolitica *biovar 1A showed clustering with members of gamma-proteobacteria such as *P. luminescens *and *E. ictaluri *along with *Yersinia *spp. (See Additional files 2 and 3). These protein sequences were also related closely to members of alpha-proteobacteria like *Methylobacterium chloromethanicum*, *M. extorquens*, *M. populi *and *Brucella *spp. but were related distantly to other members of gamma-proteobacteria like *Klebsiella aerogenes*, *P. mirabilis *and *Escherichia coli*.

### PCR-RFLP of ure genes

**Figure 2:**
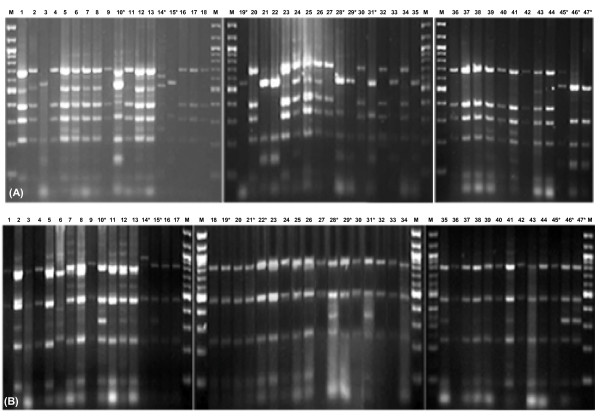
**PCR-RFLP of *ureC***. PCR-RFLP of *ureC *of *Y. enterocolitica *biovar 1A strains amplified with primers ureC1-ureC4, and restriction digested using (A) *Rsa*I and (B) *Sau*96I enzymes. Lanes 1: IP27360, 2: IP27362, 3: IP27364, 4: IP27365, 5: IP26310, 6: IP26311, 7: IP26312, 8: IP26315, 9: IP27403, 10: IP27407, 11: IP27429, 12: IP27433, 13: IP27434, 14: IP26261, 15: IP26305, 16: E1281580, 17: IP26316, 18: E1281550, 19: IP26152, 20: P346, 21: P354, 22: P386, 23: P472, 24: IP27404, 25: IP27406, 26: IP27430, 27: IP27432, 28: IP27484, 29: IP26147, 30: IP26148, 31: E1281600, 32: IP27385, 33: IP27386, 34: IP27388, 35: IP27485, 36: STM 126, 37: 8660/90 STM 484, 38: 0310/90, 39: ST5 NF-O, 40: IP27879, 41: IP27873, 42: IP27950, 43: IP27985, 44: IP24121, 45: IP27648, 46: IP27210, 47: IP27149, M: Molecular mass marker (100 bp ladder, New England BioLabs). * Strains belonging to clonal group B are shown in lanes 10, 14, 15, 19, 21, 22, 28, 29, 31, 45, 46 and 47. Clonal group A strains are in other lanes. For clonal groups refer to [22].

The regions constituting the structural genes namely *ureAB *and *ureC *were amplified in several *Y. enterocolitica *biovar 1A strains using primer pairs AB3-AB4 and C1-C4 respectively. Restriction digestion of *ureAB *region with *Hae*III and *Sau*96I resulted in almost identical patterns among all biovar 1A strains (See Additional file 4). But, differences were clearly evident in restriction profiles of *ureC *digested with *Rsa*I and *Sau*96I (Fig. 2). With *Rsa*I, strains belonging to clonal group A exhibited profile different from that of clonal group B strains. Thus, it may be inferred that sequence of urease gene in clonal group A strains is different from that of clonal group B strains.

The *Hae*III and *Sau*96I restriction profiles of *ureAB *of biovar 1B, 2 and 4 strains were distinct from that of biovar 1A strains (See Additional file 4). As with *ureAB*, restriction patterns of *ureC *for these biovars were also quite distinct from biovar 1A strains (data not shown).

### Biochemical characterization

**Figure 3:**
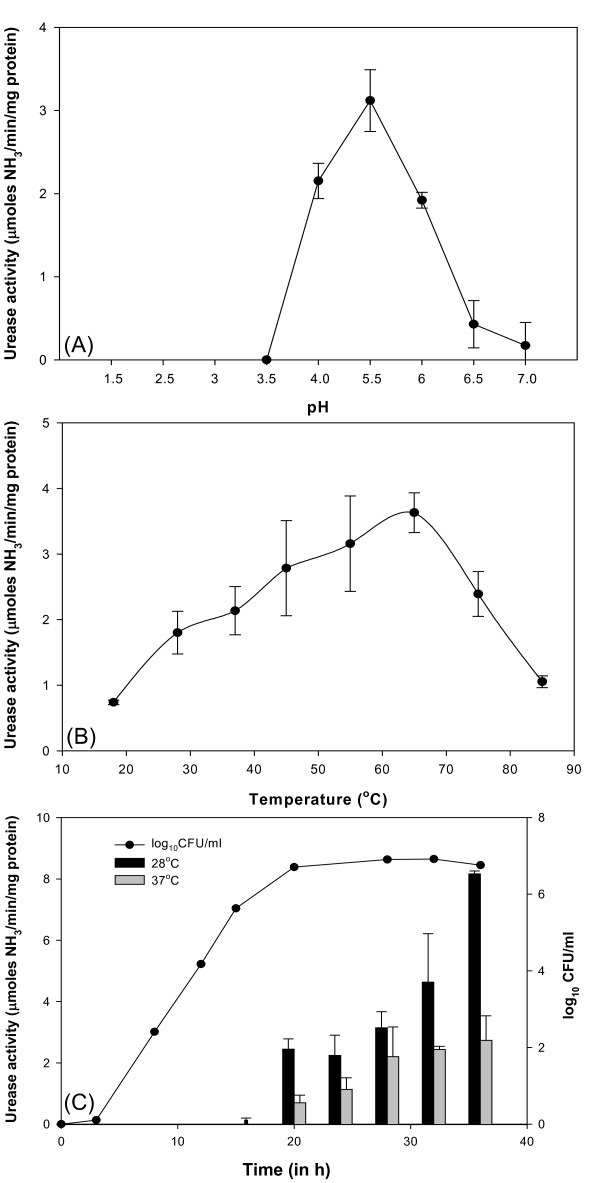
**Biochemical characterization of *Y. enterocolitica *biovar 1A urease**. (a) optimal pH for urease activity (b) effect of temperature on urease activity and (c) effect of growth phase and growth temperature on urease production; growth curve of biovar 1A strain grown at 28°C is also shown. Data points represent mean of triplicate determinations. The error bars indicate standard deviation.

The crude extract of urease of *Y. enterocolitica *biovar 1A strain was active over a pH range of 4.0-7.0. The maximum activity was observed at pH 5.5 (Fig. 3a). The enzyme was quite heat-stable as urease activity was recorded up to 65°C but decreased progressively at higher temperature (Fig. 3b). The optimum temperature for urease activity was 65°C (Fig. 3b). The urease exhibited Michaelis-Menten kinetics with K_m _and V_max _of 1.74 ± 0.4 mM urea and 7.29 ± 0.42 μmol of ammonia released/min/mg of protein respectively (data not shown).

*Y. enterocolitica *biovar 1A grown at 28°C (optimum temperature for growth) exhibited higher urease activity than that grown at 37°C (Fig. 3c). Irrespective of the growth temperature, stationary phase cells showed higher activity (Fig. 3c). The supplementation of growth medium (Luria broth) with 16.7 mM urea did not show significant difference in urease activity. However, supplementation with nickel chloride resulted in *ca*. 10-fold increase in the activity. 1 μM NiCl_2 _was sufficient to induce urease activity as no significant increase in the activity was observed with further increase in concentration up to 200 μM (See Additional file 5).

**Figure 4:**
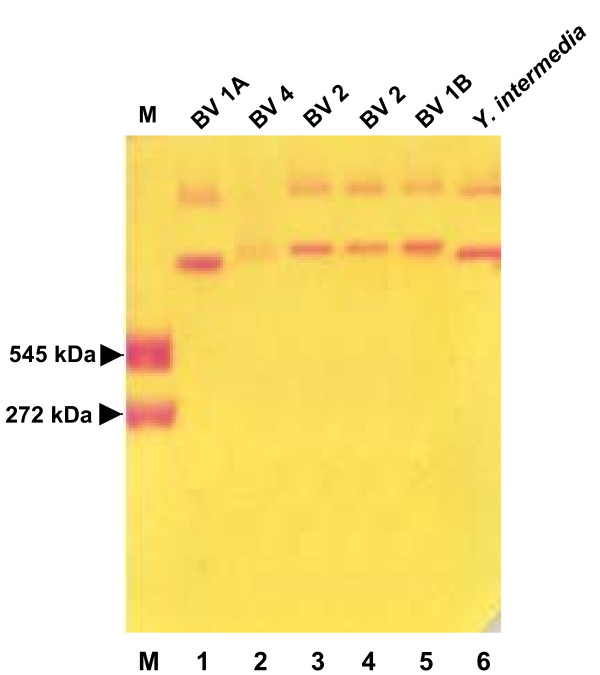
**Non-denaturing PAGE showing urease activity of *Yersinia *spp. **Lane 1: *Y. enterocolitica *IP27403 (1A/O:6,30); lane 2: *Y. enterocolitica *IP134 (4/O:3); lane 3: *Y. enterocolitica *IP26329 (2/O:9); lane 4: *Y. enterocolitica *IP26249 (2/O:5,27); lane 5: *Y. enterocolitica *8081 (1B/O:8); lane 6: *Y. intermedia *IP27478 (serotype O:7,8-8); M: Jack bean urease [272 kDa (trimer) and 545 kDa (hexamer); BV: Biovar.

On native PAGE, urease was observed as two bands with the major band having molecular weight > 545 kDa and a slowly-developing band above it (Fig. 4). The electrophoretic mobility of urease of *Y. enterocolitica *biovar 1A strain was shown to be different from that of biovar 1B, 2 and 4 strains though similar to the *Y. intermedia *urease. The isoelectric point of the crude extract urease was 5.2.

### Survival of *Y. enterocolitica in vitro*

**Figure 5:**
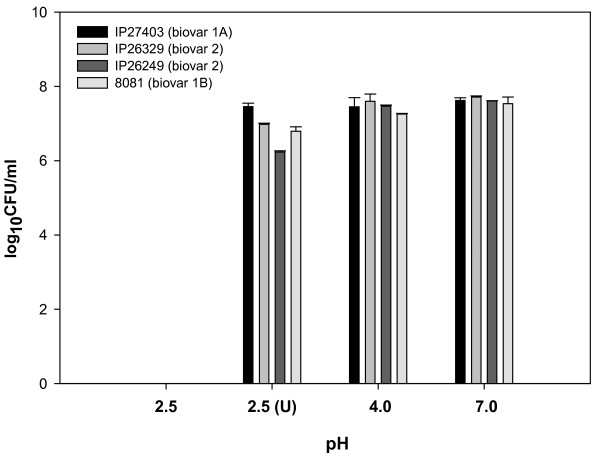
**Survival of *Y. enterocolitica in vitro *at different pH**. Number of bacterial cells (log_10_CFU/ml) of *Y. enterocolitica *after incubation for 2 h at pH 2.5, 4.0 and 7.0 in the absence and presence (U) of 3.4 mM urea. The values are mean of three independent observations. The error bars indicate standard deviation.

The ability of *Y. enterocolitica *biovar 1A strain to survive at pH 2.5, 4.0 and 7.0 *in vitro *was investigated. Strains belonging to other biovars were also studied concurrently. The biovar 1A strain survived at pH 4.0 and 7.0 for 2 h without significant differences in their viable counts (Fig. 5). However, no viable cells were recovered after 2 h at pH 2.5. In fact, the decrease in the viable counts at this pH was evident even within 5 min of incubation. The addition of 3.4 mM urea at pH 2.5 was sufficient to increase the survival of *Y. enterocolitica *biovar 1A equivalent to that observed at pH 4.0 and 7.0. Similar results were observed for other biovars also. The pH of the assay medium at the end of experiment was same as that at the start, suggesting that increased survival of *Y. enterocolitica *was not due to any significant change in the pH.

## Discussion

The *ure *gene cluster of *Y. enterocolitica *biovar 1A strain included three structural (*ureA*, *ureB*, *ureC*) and four (*ureE*, *ureF*, *ureG*, *ureD*) accessory genes. The *yut *gene, which is required for transport of urea was present downstream of this cluster. Thus, the organization (*ureABCEFGD*) of *ure *gene cluster in *Y. enterocolitica *biovar 1A strain was similar to that reported for *Y. enterocolitica *biovar 1B, *P. luminescens *and *E. ictaluri *[23,36,37]. Similar organization has been reported for other species such as *Streptococcus salivarius*, *Synechococcus *sp. WH7805, and *B. abortus ure*-2 operon [19,38,39]. However, important differences were observed compared to urease genes of *Y. enterocolitica *biovar 1B and biovar 4 strains. These included differences in the size of *ureB *gene and the intergenic regions. Also, the restriction profiles of *ure *structural genes of biovar 1A strains were different from that of biovars 1B, 2 and 4. These observations indicated that RFLP of urease genes may be used to study the epidemiology of *Y. enterocolitica*.

The amino acid residues in the urease structural proteins namely UreA (γ subunit), UreB (β subunit) and UreC (α subunit) that are reported to have functional significance in *K. aerogenes *urease [40] were also conserved in *Y. enterocolitica *biovar 1A. The crystallographic [41] and genetic [40] analysis of *K. aerogenes *urease has shown that four histidine residues (His-134, -136, -246 and -272), an aspartate (Asp-360) and a carbamylated lysine (Lys-217) of UreC are involved in nickel metallocenter binding. All these amino acids were conserved at positions His-139, -141, -251, -277; Asp-365 and Lys-222 in UreC of *Y. enterocolitica *biovar 1A. Histidine residues in the α-subunit of *K. aerogenes *shown to be important for substrate binding (His-219) and catalysis (His-320) are present at positions 224 and 325 in α-subunit of biovar 1A [40]. The urease active-site consensus sequence (MVCHHLD) [42] deviated by two residues (MVCHNLN) in biovar 1A strain. Amino acid residues with functional significance including His-97 (UreA) and His-39, -41 (UreB) [40] were also conserved in relative positions in *Y. enterocolitica *biovar 1A. The conservation of amino acids in *Y. enterocolitica *biovar 1A urease involved in coordination of nickel at active site, substrate binding and catalysis as seen in *K. aerogenes *urease, suggested similar quaternary structure of the two enzymes. UreE consisted of histidine-rich motif at carboxy terminus as in UreE of *K. aerogenes*, *B. abortus*, *Actinobacillus pleuropneumoniae, E. ictaluri *and *Synechococcus *[19,36,39,43,44]. A P-loop motif (GPVGSGKT), which contains ATP and GTP binding sites [45] and probably provides energy for Ni activation [46] was present at the amino terminus (positions 19-26) of UreG.

A pH optimum in the acidic range for urease produced by a neutrophile like *Y. enterocolitica *biovar 1A was similar to that reported for *Y. enterocolitica *biovars 1B and 4, and *Morganella morganii *[35,47]. Ureases with optima in the acidic range reportedly carried a phenylalanine seven residues towards N-terminus, and an asparagine one residue toward the C-terminus, from the catalytic site [35]. Both these residues are also present at respective positions in UreC of *Y. enterocolitica *biovar 1A. The maximal activity of urease at 65°C by *Y. enterocolitica *biovar 1A has also been reported for other bacteria [44]. A low K_m _of *Y. enterocolitica *biovar 1A urease as in biovar 4 strains [47], indicated its high affinity for urea. This suggested that the enzyme might function quite normally in the gut despite low concentrations (1.7-3.4 mM) of the urea available there. Also, consistent with our observation, organisms which produce urease with low K_m _have been reported to possess urea transport (*yut*) gene as seen in *S. salivarius*, *Lactobacillus fermentum*, *Bacillus *sp. strain TB-90 and *B. suis *[48].

The cultural conditions which affected production of urease by *Y. enterocolitica *biovar 1A included growth phase, growth temperature and availability of nickel ions. The expression of bacterial ureases is known to be either constitutive or induced by factors like low nitrogen, urea or pH [49]. The maximal urease activity during stationary phase of the growth and at 28°C as observed for *Y. enterocolitica *biovar 1A strain was consistent with that of biovar 4 strains [47]. In *Y. enterocolitica*, several other virulence factors such as invasin, Myf fibrillae and enterotoxin have also been reported to be regulated by growth phase and the growth temperature [50]. A 10-fold increase in urease activity following supplementation of growth medium with nickel was not accompanied by increase in the expression of urease structural proteins suggesting that increased activity was probably due to the activation of pre-existing apoenzyme. Nickel has been reported to regulate both expression and activity of urease in *H. pylori *[51]. *In silico *analysis of whole genome of *Y. enterocolitica *8081 (biovar 1B) revealed two systems (*ureH *and *ynt*) for transport of nickel. It would be interesting to determine the role of multiple nickel transport genes in urease activity and its regulation in *Y. enterocolitica*.

The M_w _of *Y. enterocolitica *biovar 1A urease as assessed from native PAGE was > 545 kDa. The molecular mass of urease is known to vary from as low as 130 kDa in *B. suis *[52] to as high as 620 kDa in *Providencia rettgeri *or > 700 kDa in *M. morganii *[53]. The difference in the molecular mass of urease of *Y. enterocolitica *biovar 1A *vis-à-vis Y. enterocolitica *biovar 1B and biovar 4 seems to be due to difference in the size of UreB (β-subunit), which is smaller in the former and thus may account for its lower molecular mass. The isoelectric point (pI) of 5.2 of biovar 1A urease was close to that reported for *Proteus penneri *(pI = 5.1) and *H. pylori *(pI = 5.9) urease [33,54]. No data on molecular mass and isoelectric point of ureases produced by *Y. enterocolitica *strains belonging to other biovars has been reported.

The ability of *Y. enterocolitica *biovar 1A strains to survive at pH 2.5 *in vitro *in the presence of 3.4 mM urea implicated urease in their survival. This suggested the possible role urease might play in the survival of *Y. enterocolitica *biovar 1A under acidic conditions in the gut. However, this needs to be confirmed by comparison of wild type strain with an isogenic urease mutant. The role of urease in survival during transit through gut has been reported for *B. suis*, *B. abortus*, *H. pylori *and *E. ictaluri *[18,19,36,55,56]. Interestingly, the biovar 1A strains have also been reported to resist killing, and survive within macrophages [13]. It would therefore be worthwhile to determine the role urease may play in the survival of *Y. enterocolitica *biovar 1A strains in the acidic environment of phagolysosomes.

## Conclusions

The *ure *gene cluster of *Y. enterocolitica *biovar 1A though broadly similar to that of biovar 1B and biovar 4 strains showed differences in structural (*ureB*) genes and the intergenic regions thereof. The kinetic data indicated that urease produced by *Y. enterocolitica *biovar 1A strain would be active at low concentration of urea typically present in the gut. The ability of biovar 1A strain to survive at acidic pH in the presence of urea suggested that urease might play role in their survival in the gut. This however needs to be corroborated using *ure *isogenic mutants.

## Authors' contributions

NB carried out the experimental part of the study. JSV conceived and supervised the work. Both authors participated in interpretation of data and preparation of the final manuscript.

## Supplementary Material

Additional file 1 **Nucleotide and deduced amino acid sequences of *ure *gene cluster of *Y. enterocolitica *biovar 1A**. The nucleotide sequence of the *ure *gene cluster of *Y. enterocolitica *biovar 1A and the deduced amino acid sequences of the structural (A, B, and C) and accessory (E, F, G and D) proteins are shown. Putative ribosome binding site consensus sequences upstream of *ureA*, *ureB*, *ureC*, *ureF *and *ureG *are in bold face. Stop codons are indicated by an asterisk. Click here for file

Additional file 2 **Phylogenetic relationships of urease structural (UreA, UreB and UreC) proteins**. Dendrograms showing phylogenetic relationships of *Yersinia *spp. including *Y. enterocolitica *biovar 1A and other bacterial species based on amino acid sequence of urease structural proteins (UreA, UreB and UreC). The trees were constructed by the neighbor joining method in MEGA 4.0 package. The bootstrap values presented at corresponding branches were evaluated from 1,000 replications. GenBank accession numbers are indicated for strains used in creating the dendrogram. The bar scale shows substitutions per site. Click here for file

Additional file 3 **Phylogenetic relationships of urease accessory (UreE, UreF UreG and UreD) proteins**. Dendrograms showing phylogenetic relationships of *Yersinia *spp. including *Y. enterocolitica *biovar 1A and other bacterial species based on amino acid sequence of urease accessory proteins (UreE, UreF, UreG and UreD). The trees were constructed by the neighbor joining method in MEGA 4.0 package. The bootstrap values presented at corresponding branches were evaluated from 1,000 replications. GenBank accession numbers are indicated for strains used in creating the dendrogram. The bar scale shows substitutions per site. Click here for file

Additional file 4 **PCR-RFLP of *ureAB *of *Y. enterocolitica***. DNA was amplified with primers ureAB3-ureAB4 and restriction digested using (A) *Hae*III and (B) *Sau*96I enzymes. Lanes 1: IP27403, 2: IP26305, 3: E1281550, 4: P346, 5: P472, 6: IP27387, 7: STM 126, 8: 0310/90, 9: IP27938, 10: IP27879, 11: IP27873, 12: IP24121, 13: IP134, 14: IP26329, 15: IP26249, 16: 8081. M: Molecular mass marker (100 bp ladder, New England BioLabs); BV: biovar. Click here for file

Additional file 5 **Effect of urea/nickel chloride on activity (A) and expression (B) of urease of *Y. enterocolitica***. Strain IP27403 was grown in Luria Broth (LB) or in LB supplemented with 16.7 mM urea (LB-urea) or NiCl_2 _at 1 μM (Ni-1), 10 μM (Ni-10), 100 μM (Ni-100) and 200 μM (Ni-200) concentration. M: Medium range protein ladder (Bangalore Genei). Data points represent mean of triplicate determinations; error bars denote standard deviation. Click here for file
