# Supplementary material for: Molecular and biochemical characterization of urease and survival of Yersinia enterocolitica biovar 1A in acidic pH in vitro
Source: BMC Microbiol. 2009 Dec 17;9:262. doi: 10.1186/1471-2180-9-262 (PMC2806259; doi:10.1186/1471-2180-9-262)
Supplement: Additional file 1 — Nucleotide and deduced amino acid sequences of ure gene cluster of Y. enterocolitica biovar 1A. The nucleotide sequence of the ure gene cluster of Y. enterocolitica biovar 1A and the deduced amino acid sequences of the structural (A, B, and C) and accessory (E, F, G and D) proteins are shown. Putative ribosome binding site consensus sequences upstream of ureA, ureB, ureC, ureF and ureG are in bold face. Stop codons are indicated by an asterisk. [file 1471-2180-9-262-S1.PDF]

ggagggcttatgcagctcaccccaagagaagttgaaaagctcatgatctacacgctgtctgatgtggcgttcaaacgcaaagcgcgtggcttgaaactca 100
ureA M Q L T P R E V E K L M I Y T L S D V A F K R K A R G L K L

attatccggaagccgtttctattatcacagtgactgcaatggaaggggccagagatggcaaatccgtagaggatgtgatgaaagaagccagtaaagttct 200
N Y P E A V S I I T V T A M E G A R D G K S V E D V M K E A S K V L

cacaaaagatgatgtgatggacggggtggctgatctgattccgaatgttcaggtcgaagcaatttttaccgacggtagccgtttggtcacagtgcacgac 300
T K D D V M D G V A D L I P N V Q V E A I F T D G S R L V T V H D

cctatcaaatgagtgacggcaacatgcgagtaaaaacctgaactgaattttgca**gagg**atcaaagcatgagcgcaaagaaaagcactaaagataatgcag 400
P I K * *ureB* M S A K K S T K D N A

aacaaaataccccattgggtggttgcattttagccgatacaccgatcacctttaatgaaaacaagcccgttaccaaagtaaaagttcgcaataccggtga 500
E Q N T P L G G C I L A D T P I T F N E N K P V T K V K V R N T G D

ccggccaattcaggtgggctcacatttccacttctttgaagctaaccgggcactggagtttgaccgtgctgcggcttacggaaaaaggctgaatatctct 600
R P I Q V G S H F H F F E A N R A L E F D R A A A Y G K R L N I S

tcaacaaccgccatccgttttgaacccggtgatgaaaccgaagttccgctgattccttttggtggtaagcaaacactatatggctttaacaacctggtgg 700
S T T A I R F E P G D E T E V P L I P F G G K Q T L Y G F N N L V

atggttggaccggtgaaggcgtcgttcccaatagcgaacgtccggataagctagaggctattcgtcgtgcggctgaacgtggtttcaaatcgtctaaatg 800
D G W T G E G V V P N S E R P D K L E A I R R A A E R G F K S S K *

acaccttacctaactaaaaatcatcgaaattcgcatacgtgtaattagctataaccaatagatttcaagtttcaggaaggcggcaaacgagagagttccg 900

atgagcttacaccggtaagtgattcgggtgagcgagtgtagctaacacacctgcaacttgaaagatgaaggttacgcgtaccggaaaaagaa**aggag**cga 1000
ureC

cagatgcctcaaatttctcggcaagaatacgcgggtctatttggcccaacgactggcgataaaatccgtttgggtgacaccaatctgtttatcgaaatcg 1100
M P Q I S R Q E Y A G L F G P T T G D K I R L G D T N L F I E I

aaaaagacctgcgtggatatggtgaagagtcggtttacggtgggggcaagtcattgcgtgacgggatgggcgcgaataaccatctgacccgcgataacgg 1200
E K D L R G Y G E E S V Y G G G K S L R D G M G A N N H L T R D N G

tgtactggatttagtcataaccaacgtcactatcgttgatgctcgtttaggggttatcaaagccgacgtcggtatccgtgatggtaaaattgccggtatc 1300
V L D L V I T N V T I V D A R L G V I K A D V G I R D G K I A G I

ggtaaaagtggtaacccaggggtgatggatggcgtgactcccggcatggtcgtgggcgtaagcaccgacgctatttccggtgagcatttgattctgaccg 1400
G K S G N P G V M D G V T P G M V V G V S T D A I S G E H L I L T A

ccgccggtattgatagccacattcacttaatctccccacaacaggcttatcatgcgctatctaatggcgtggcaaccttctttggtggtgggattggccc 1500
A G I D S H I H L I S P Q Q A Y H A L S N G V A T F F G G G I G P

aaccgacggtactaatgggactacagtgacacctggcccttggaatattcgtcagatgttgcgctcagttgaagggctgccagtcaacgtgggtattctg 1600
T D G T N G T T V T P G P W N I R Q M L R S V E G L P V N V G I L G

ggtaaaggtaactcttatggccgtggcccgctgttggaacaggcgattgccggtgttgtcggctataaagttcacgaagactggggcgcgacagctaatg 1700
G K G N S Y G R G P L L E Q A I A G V V G Y K V H E D W G A T A N

ccctgctccattcattacggatggcggatgaaatggatattcaggtttccgtgcataccgacagtttgaacgaatgtggttatgtagaagacaccattga 1800
A L L H S L R M A D E M D I Q V S V H T D S L N E C G Y V E D T I D

tgccttcgaaggccgcaccatccataccttccacaccgagggtgcgggcgggggccatgcgccggacatcatccgtgttgccagccagcctaacgtacta 1900
A F E G R T I H T F H T E G A G G G H A P D I I R V A S Q P N V L

ccaagttcgactaacccaaccctgccatacggggttaacagccaggccgaactgttcgacatgatcatggtgtgtcataacctcaacccgaatgtgcctg 2000
P S S T N P T L P Y G V N S Q A E L F D M I M V C H N L N P N V P

ctgacgtctcctttgccgaaagccgtgtgcgcccggaaaccatcgcggcagaaaacgttctgcacgatatgggggttatctccatgttctccagtgactc 2100
A D V S F A E S R V R P E T I A A E N V L H D M G V I S M F S S D S

acaagccatggggcgtgttggggaaaactggctgcgtgtgatgcaaacggctaacgcaatgaaagcatcacgcggcaaattgccagaagatgcgccgggt 2200
Q A M G R V G E N W L R V M Q T A N A M K A S R G K L P E D A P G

aacgataacttccgcgtcctgcgctatgtggcaaaaatcaccataaacccagcgattgcacaaggtgtcagccatgtcatcggttcagttgaagtgggca 2300
N D N F R V L R Y V A K I T I N P A I A Q G V S H V I G S V E V G

aaatggccgatctggtgttgtgggatccacgcttctttggtgcgaaacctaagatggttatcaaaggcggcatgatcaactgggcggcaatgggggatcc 2400
K M A D L V L W D P R F F G A K P K M V I K G G M I N W A A M G D P

gaatgcctcattaccaactccacaaccggtgttctatcgtccaatgtttggcgccatgggtaaaaccatgcaagacacctgcgtcaccttcgtttctcag 2500
N A S L P T P Q P V F Y R P M F G A M G K T M Q D T C V T F V S Q

gctgcgctggatgacggtgtgaaagagaaagccgggctggatcgccaggttattgcggctaaaaactgccgtaccatttctaaacatgacctggtgcgta 2600
A A L D D G V K E K A G L D R Q V I A A K N C R T I S K H D L V R

atgaccaaacaccaaacattgaagtggaccctgaaacctttgcggtgaaagtgaatggcgtacatgccacctgtgagcctattgatacagcgtcgatgaa 2700
N D Q T P N I E V D P E T F A V K V N G V H A T C E P I D T A S M N

ccaacgctacttctttggttaataagagctttggttaataagggctttggttaataagagctttggttgataaaggctttggttaacggataaatctgga 2800
Q R Y F F G *

ctcaaatccagtttattaagcattcagtttgctcatggtaagtgtcatctggttgttggatggggcgaccaacgatggccgggatgtgtggcacagaaac 2900

ggatttcattactgtgggcaactggcttttgccaaaaatacaggcaaggagtcaatacatgattttgatagagcacattcttggcaatgtgaaaaaagat 3000
ureE M I L I E H I L G N V K K D

ccggtttggcaggagaaactcaaagacgccacttttgatctcttggttttggatcaacgggaagcgcaaaaaagccgttgccgtaaactcagcacgcagg 3100
P V W Q E K L K D A T F D L L V L D Q R E A Q K S R C R K L S T Q

ggttagatctgggtatttcgctcgaccgacacgtcgttctggctgatggagatgtgctggcgtgggatgaaaaaaccaatgtcgcggtggtggtacaaat 3200
G L D L G I S L D R H V V L A D G D V L A W D E K T N V A V V V Q I

caatttgcgcgatgtcatggttatcgatctgagtgaactgaaaagccgttcaccggatgaactgattaaaacctgctttgagctggggcacgcactgggt 3300
N L R D V M V I D L S E L K S R S P D E L I K T C F E L G H A L G

aaccagcactggaaagcagtgacgaaaaataacgaggtctatgtgcctctgacggttgccaccaccatgatggattccgtgatgagaacccacggcttcc 3400
N Q H W K A V T K N N E V Y V P L T V A T T M M D S V M R T H G F

agcatttacctttccgttttgttaaaggggcggaaattctaccgttactcagtaattctgaagcgcgcctgctatttggcggggctgaagataccgatac 3500
Q H L P F R F V K G A E I L P L L S N S E A R L L F G G A E D T D T

tcatgtgcatgtcgccagccctttggatgaacctcatggctccggcttacatgttcacgcgattcattcccacggcgatgggcatacacatagccatgac 3600
H V H V A S P L D E P H G S G L H V H A I H S H G D G H T H S H D

cacgaccacagtcatagccacggcgaccacgaccataaacactgattctggca**gggag**gcaccgcaatgaatgcatcagatctgattcgtatcatgcaat 3700
H D H S H S H G D H D H K H * *ureF* M N A S D L I R I M Q

ttggtgattccgtactgccggtcggggccttcacgttttccaatggcgtggagtccgccattcaaactggcgtggtacgcgacgtgccgacgttaaaagg 3800
F G D S V L P V G A F T F S N G V E S A I Q T G V V R D V P T L K G

cttcgtgttaaccgccttaaaacaagcggccagttgtgatggcatgggggtagttgctgcccatcgggcggtagtagccgacgatcgtgacggtattatc 3900
F V L T A L K Q A A S C D G M G V V A A H R A V V A D D R D G I I

cgtgctgattgggcggtgaataaccgcaaactcaatgaagaaagccgcctgatggcaacccgaatggggaaaaaaactggcggagatgtcaatccatgtgg 4000
R A D W A V N N R K L N E E S R L M A T R M G K K L A E M S I H V

tggagcatccgctgatcagctggtggctggaacagataaaaaatggcaataccgcagggacttacccggttactcaggcggtggtgatggccgcacaggg 4100
V E H P L I S W W L E Q I K N G N T A G T Y P V T Q A V V M A A Q G

gattgggcagcgcgaagtggtggtgatgcaccaatatggcgtggcgatgacaatattaagtgcggcgatgcgtttgatgcgcgttacccatttcgacact 4200
I G Q R E V V V M H Q Y G V A M T I L S A A M R L M R V T H F D T

cagcatatcttgtttgaattaaaccacgacatcgagaagttctgcgatattgccgaaattggcgatattgaccagatgtcttcttatgtccctattgtgg 4300
Q H I L F E L N H D I E K F C D I A E I G D I D Q M S S Y V P I V

atgttttggcggcggtgcatgtgaaagcgcacgttcgcctgtttagtaactgatcgactacttaactgattgataaaaatgacttctgagtgctgggtga 4400
D V L A A V H V K A H V R L F S N *

gtgggctcgccgaggacagcttatttgccaactacttggccaatatcagataaaactaa**gagga**ttaccccgtgaatagccattcaaccgataaacgcaa 4500
ureG V N S H S T D K R K

aaagatcacccgcattggtattggtggcccggtgggttcaggtaaaaccgccattatcgaagtgatcacccctattctgatcaaacggggtattaagccg 4600
K I T R I G I G G P V G S G K T A I I E V I T P I L I K R G I K P

ctgatcattaccaatgacatcgtcaccaccgaggatgctaaacaggtgaaacgtaccctgaaaggcattctggatgaagagaagatcctcggggtcgaaa 4700
L I I T N D I V T T E D A K Q V K R T L K G I L D E E K I L G V E

ccggtgcttgcccgcatactgcggtgcgtgaagacccaagtatgaatattgctgcggtggaagagatggaagagcgcttccctgacagcgacctcatcat 4800
T G A C P H T A V R E D P S M N I A A V E E M E E R F P D S D L I M

gattgaaagcggtggcgacaacctgacactgacctttagcccggccttggccgacttctatatctatgtcatcgatgtggcggaaggggaaaaaatcccg 4900
I E S G G D N L T L T F S P A L A D F Y I Y V I D V A E G E K I P

cgtaaaaatggcccaggtttggttcaggcggacattcttgtcatcaacaaaattgacctcgccccttatgtcggtgccagcctggatgtgatggaaagtg 5000
R K N G P G L V Q A D I L V I N K I D L A P Y V G A S L D V M E S

acaccaaagtggttcgtggcgagcgcccttatattctgaccaactgcaaaaccgggcagggcattgaagagttggtggatatgattatgcgcgacttctt 5100
D T K V V R G E R P Y I L T N C K T G Q G I E E L V D M I M R D F L

gtttacccatgtgcagccacaaggagaacatgcatgacatcgcagagccagaatatcgtggaaactccttcacgggttcgcgctcacgcattatgtctca 5200
F T H V Q P Q G E H A *
ureD M T S Q S Q N I V E T P S R V R A H A L C L

acgcgccggaactcgcggaataccaagatgaaccggcgcaaatgcgtagcggggcggtagggaaaagcggctatcttaaactgagatttgccaaacgtga 5300
N A P E L A E Y Q D E P A Q M R S G A V G K S G Y L K L R F A K R E

acatcgcagtatttgggccgaaatggaaagacgggtgccctcaatggtgcaaaaagcgctgtactgggatgaagaaatgcccgaactgccgtgtgtcacc 5400
H R S I W A E M E R R V P S M V Q K A L Y W D E E M P E L P C V T

atgatctcgacgtcaggatgcattttacaaggtgaccgtctggccactgacgtgattgtggaggcgggggcttgcgcccatgtcactacgcaatcggcga 5500
M I S T S G C I L Q G D R L A T D V I V E A G A C A H V T T Q S A

ccaaagttcatatgatgaatgccaactacgcgtcgcagatacagaattttacggtggaagagggaggggtatcttgaatttatgccagacccacttattcc 5600
T K V H M M N A N Y A S Q I Q N F T V E E G G Y L E F M P D P L I P

acatcgtaattcgcgttttattactgataccaccattaatattcatcctacggcgacggcgatttattcggaagtgctgatgtctgggcgtaaatatcac 5700
H R N S R F I T D T T I N I H P T A T A I Y S E V L M S G R K Y H

catgcggacgaacgctttggttttgatgtttattcctcccgagtggctgcgcacgtttttttgggtaaagaacagccagcaggtaaagagctgtttgttg 5800
H A D E R F G F D V Y S S R V A A H V F L G K E Q P A G K E L F V

agaaatatgtgttggaaccgaagtcagaaagtcttgatgccattggggtaatgcaatcatttgatgcgttcggcaatgtgatcttgttaacccccaaaga 5900
E K Y V L E P K S E S L D A I G V M Q S F D A F G N V I L L T P K E

gcatcatgatcgcattctggcgcgggtaccggcccactttgatattaaaggcggtattgccagtggggcaacgcgcttaccaaatgattgcgggctggta 6000
H H D R I L A R V P A H F D I K G G I A S G A T R L P N D C G L V

tttaaagcactgggaatcgatagtgccggtgtgaaaaatgaaattcgacagttttggaaaatagctcgtgaggaaattctcggtgtgacattgccggaaa 6100
F K A L G I D S A G V K N E I R Q F W K I A R E E I L G V T L P E

Aattcttgtggcgctagggtgttgctcaaagttattggcgttgcagcaaaacatcgctgcaacaataaataggtgcaaagacgagtgagtgcagctaata 6200
K F L W R *

cagctgtggcttcaagttagaagggtaaaataatgaacgcgaaaacgggcaatcaatccggctgggcacaacttagtgcttcgaatgttttcattgaatt 6300
yut M N A K T G N Q S G W A Q L S A S N V F I E F

tatcgatacaacgctgcgcggctgtgctcaagtcatgtttcagaataaccctctgactgggctattcttttttatcgcaatatttgtcagagcctatggc 6400
I D T T L R G C A Q V M F Q N N P L T G L F F F I A I F V R A Y G

gaagggaatccggcagtggcttacggttgtgtgctgggaacagtagttgcaacactcaccggggctaacgatgcgcgaccgtaaatcatggcgctcagggt 6500
E G N P A V A Y G C V L G T V V A T L T G L T M R D R K S W R S G

tatacggctataacgggtgtttggtgggggcggcattaccgacatttttggttgctacaccgatagtctgggcgtgtattgttttaggtagcatcgtgtc 6600
L Y G Y N G C L V G A A L P T F L V A T P I V W A C I V L G S I V S

ggtcattgttatggtttgtatcgccgatattttaaaaacctggaaagttgcggcattaactgcaccttttgttctcacaacctggatgatcttgctggct 6700
V I V M V C I A D I L K T W K V A A L T A P F V L T T W M I L L A

agttacgcctttgccggtttacatagcagtggattacctactccggctctacctcatccgttggttttagatagtggagcaactgcgggggggtaatcttt 6800
S Y A F A G L H S S G L P T P A L P H P L V L D S G A T A G G N L

tcgtcagcatgttcaatggtgtttctcaggtatttttattcagtagtttgattggcggcattctttttatcatcggtctggcggttgaatcgctctgggc 6900
F V S M F N G V S Q V F L F S S L I G G I L F I I G L A V E S L W A

cgccgtatttgccgtaggtggttcactattggcgcttttcaccgctatattccttggggcaaacccgagcagtattgatgccggactttatgcattcagt 7000
A V F A V G G S L L A L F T A I F L G A N P S S I D A G L Y A F S

gcggtattgacagcgatcgctttaggttcgacatttaacaagcctagttggcgcgttttggcttataccatcgtcggtgttatttttactgtcatcgtgc 7100
A V L T A I A L G S T F N K P S W R V L A Y T I V G V I F T V I V

agggcgctttgaatattctattgtcccctatcggtattccaaccttgacgatgccatttgttctcgcttcatggttattc 7180
Q G A L N I L L S P I G I P T L T M P F V L A S W L F
