# Supplementary figures and images for: Molecular and biochemical characterization of urease and survival of Yersinia enterocolitica biovar 1A in acidic pH in vitro
Source: BMC Microbiol. 2009 Dec 17;9:262. doi: 10.1186/1471-2180-9-262 (PMC2806259; doi:10.1186/1471-2180-9-262)

# UreA

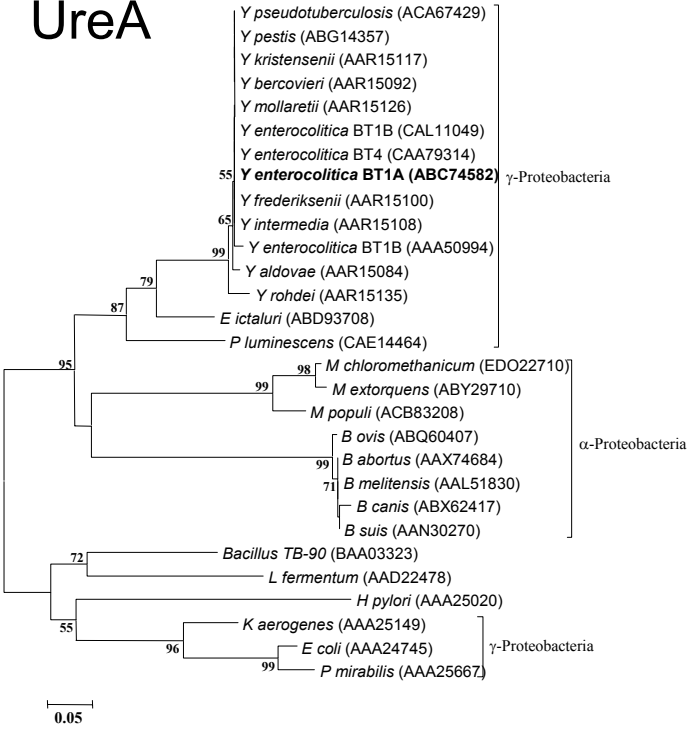

# UreB

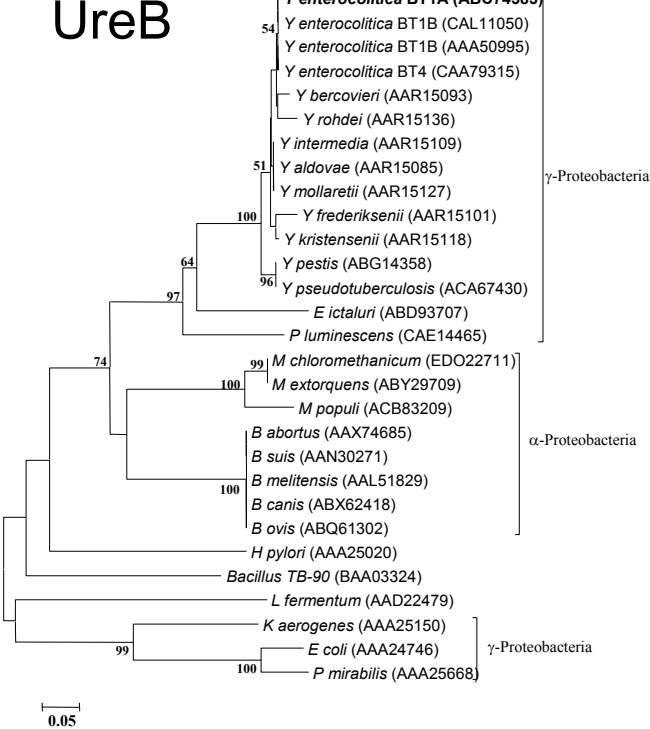

# UreC

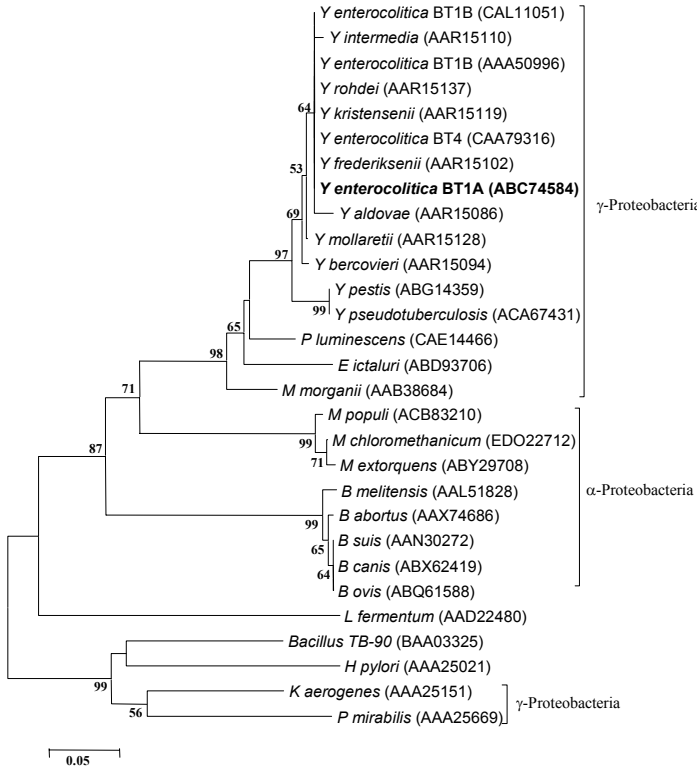

Supplement: Additional file 2 — Phylogenetic relationships of urease structural (UreA, UreB and UreC) proteins. Dendrograms showing phylogenetic relationships of Yersinia spp. including Y. enterocolitica biovar 1A and other bacterial species based on amino acid sequence of urease structural proteins (UreA, UreB and UreC). The trees were constructed by the neighbor joining method in MEGA 4.0 package. The bootstrap values presented at corresponding branches were evaluated from 1,000 replications. GenBank accession numbers are indicated for strains used in creating the dendrogram. The bar scale shows substitutions per site. [file 1471-2180-9-262-S2.PDF]

# UreE

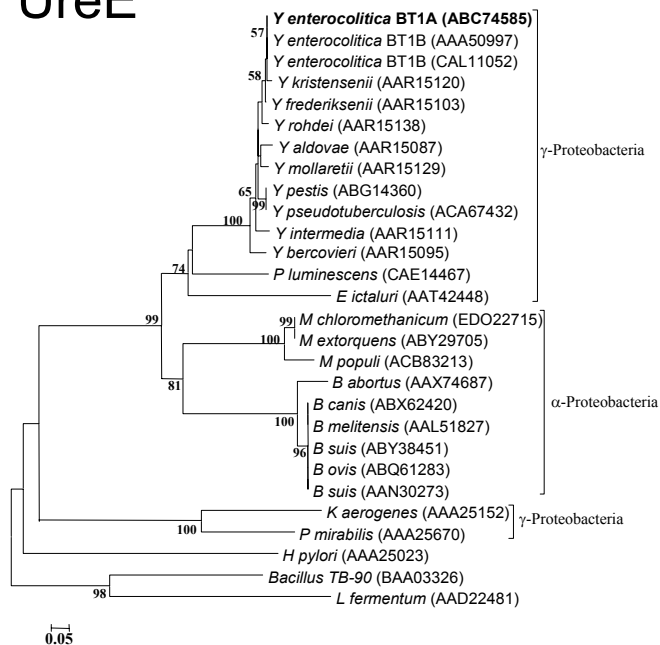

# UreF

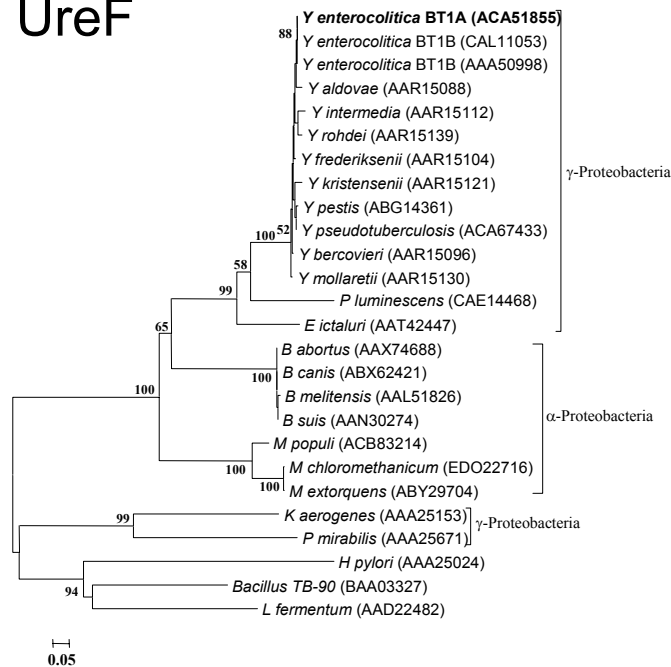

# UreG

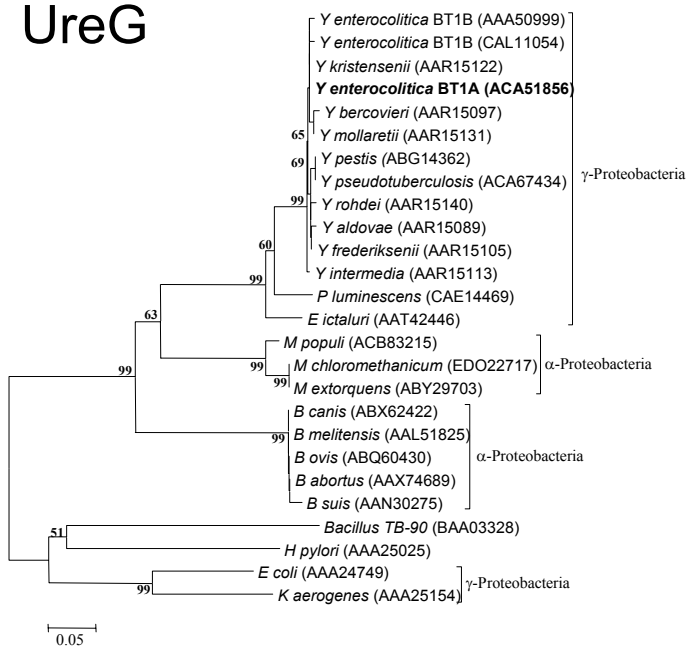

# UreD

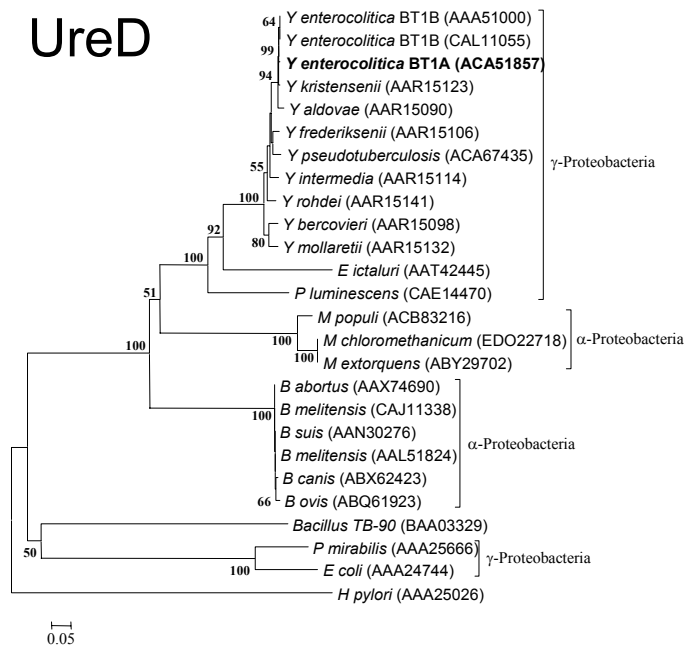

Supplement: Additional file 3 — Phylogenetic relationships of urease accessory (UreE, UreF UreG and UreD) proteins. Dendrograms showing phylogenetic relationships of Yersinia spp. including Y. enterocolitica biovar 1A and other bacterial species based on amino acid sequence of urease accessory proteins (UreE, UreF, UreG and UreD). The trees were constructed by the neighbor joining method in MEGA 4.0 package. The bootstrap values presented at corresponding branches were evaluated from 1,000 replications. GenBank accession numbers are indicated for strains used in creating the dendrogram. The bar scale shows substitutions per site. [file 1471-2180-9-262-S3.PDF]

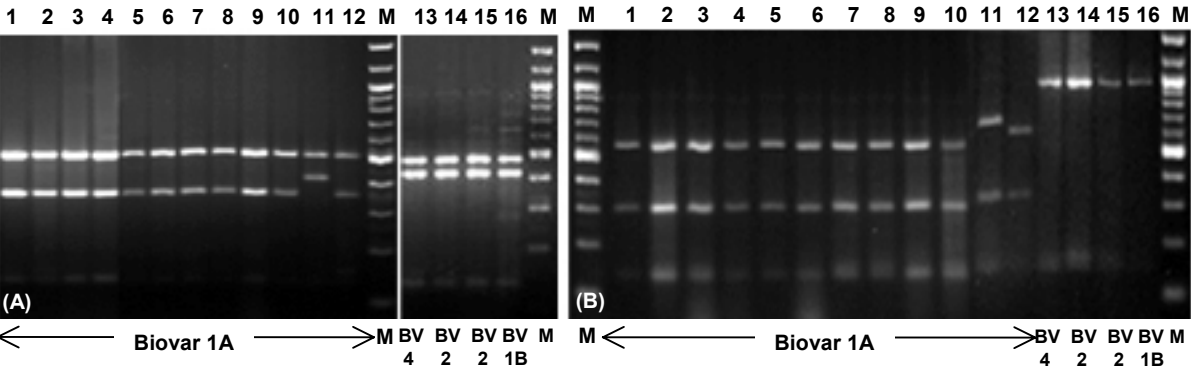

Supplement: Additional file 4 — PCR-RFLP of ureAB of Y. enterocolitica. DNA was amplified with primers ureAB3-ureAB4 and restriction digested using (A) HaeIII and (B) Sau96I enzymes. Lanes 1: IP27403, 2: IP26305, 3: E1281550, 4: P346, 5: P472, 6: IP27387, 7: STM 126, 8: 0310/90, 9: IP27938, 10: IP27879, 11: IP27873, 12: IP24121, 13: IP134, 14: IP26329, 15: IP26249, 16: 8081. M: Molecular mass marker (100 bp ladder, New England BioLabs); BV: biovar. [file 1471-2180-9-262-S4.PDF]
