# Supplementary material for: Molecular and biochemical characterization of urease and survival of Yersinia enterocolitica biovar 1A in acidic pH in vitro
Source: BMC Microbiol. 2009 Dec 17;9:262. doi: 10.1186/1471-2180-9-262 (PMC2806259; doi:10.1186/1471-2180-9-262)
Supplement: Additional file 5 — Effect of urea/nickel chloride on activity (A) and expression (B) of urease of Y. enterocolitica. Strain IP27403 was grown in Luria Broth (LB) or in LB supplemented with 16.7 mM urea (LB-urea) or NiCl2 at 1 μM (Ni-1), 10 μM (Ni-10), 100 μM (Ni-100) and 200 μM (Ni-200) concentration. M: Medium range protein ladder (Bangalore Genei). Data points represent mean of triplicate determinations; error bars denote standard deviation. [file 1471-2180-9-262-S5.PDF]

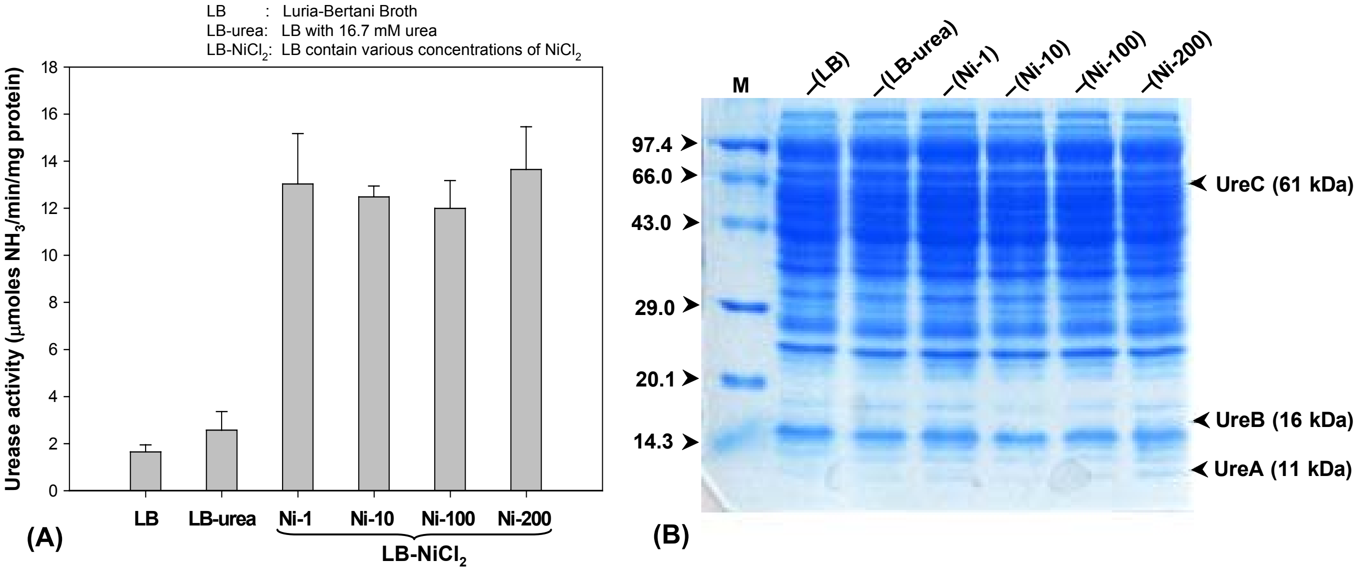

LB : Luria-Bertani Broth
LB-urea: LB with 16.7 mM urea
LB-NiCl2: LB contain various concentrations of NiCl2
Urease activity (μmoles NH3/min/mg protein)
18
16
14
12
10
8
6
4
2
0
LB
LB-urea
Ni-1
Ni-10
Ni-100
Ni-200
LB-NiCl2
(A)
M
(LB)
(LB-urea)
(Ni-1)
(Ni-10)
(Ni-100)
(Ni-200)
97.4
66.0
43.0
29.0
20.1
14.3
UreC (61 kDa)
UreB (16 kDa)
UreA (11 kDa)
(B)
